# Supplementary material for: ZNF768 Expression Associates with High Proliferative Clinicopathological Features in Lung Adenocarcinoma
Source: Cancers (Basel). 2021 Aug 17;13(16):4136. doi: 10.3390/cancers13164136 (PMC8391643; doi:10.3390/cancers13164136)
Supplement: Supplementary file 1 [file cancers-13-04136-s001.zip › cancers-1282334-supplementary.pdf]

cancers

MDPI

Supplementary Files

ZNF768 Expression Associates with High Proliferative Clinicopathological Features in Lung Adenocarcinoma

Audrey Poirier, Andréanne Gagné, Philippe Laflamme, Meagan Marcoux, Michèle Orain, Sophie Plante, David Joubert, Philippe Joubert and Mathieu Laplante

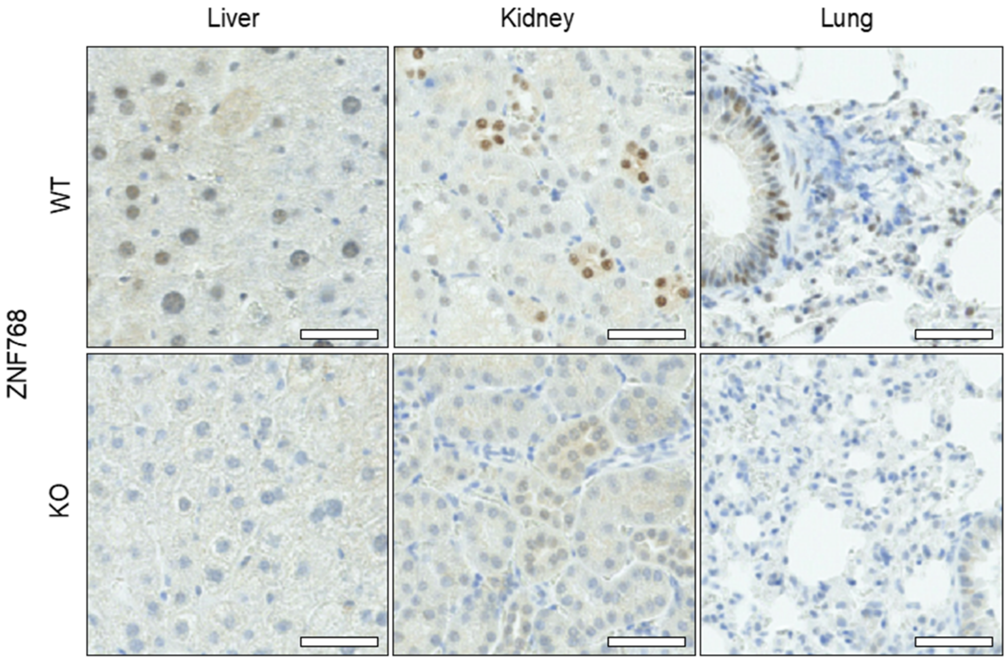

Figure S1. Knockout validation of ZNF768 antibody by immunohistochemistry in mouse tissues. Representative pictures of ZNF768 immunohistochemistry staining in different tissues of *Znf768* wild-type and knockout mice (magnification × 40, scale bar 50 μm).

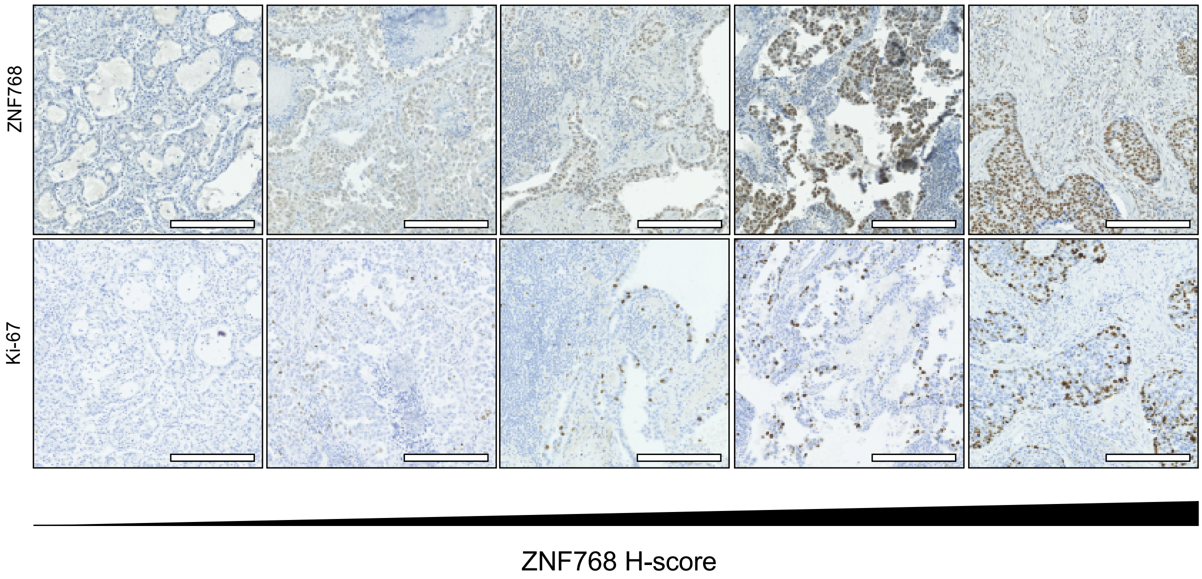

Figure S2. ZNF768 levels positively correlate with the proliferation marker Ki-67 in lung adenocarcinoma. Images of ZNF768 and Ki-67 immunohistochemistry staining in several tumor sample (magnification ×10, scale bar 250 µm). Representative samples of the correlation are shown.

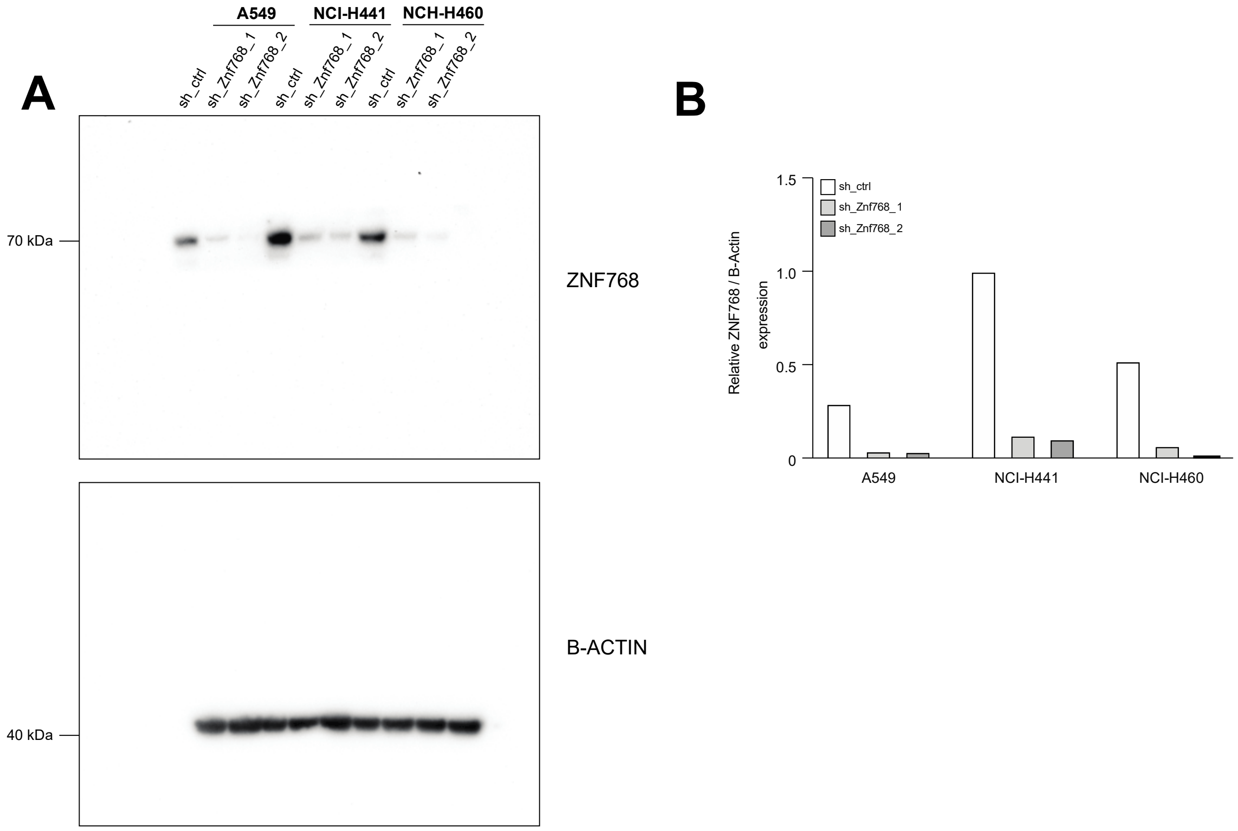

Figure S3. ZNF768 knockdown in non-small cell lung cancer cell lines. (**A**) A549 cells, NCI-H441 and NCI-H460 cells were infected with lentivirus expressing shRNA targeting ZNF768. Cells were selected with puromycin and protein were extracted. Western blot analyses were performed to confirm ZNF768 depletion (**B**) Densitometry of the western blot presented in (**A**) using B-ACTIN as a normalization control. (Supplementary data for Figure 3A–C).
